# *“Are we genuinely going to have our voices heard?”* The experience of co-producing a blended intervention to prevent relapse in obsessive-compulsive disorder: a qualitative study on the perspectives of experts by lived experience

**DOI:** 10.1186/s12888-024-06355-1

**Published:** 2024-12-18

**Authors:** Josie F.A. Millar, Nina Higson-Sweeney, Tom A. Jenkins, Erin F. Waites, Sophie Minns

**Affiliations:** Department of Psychology, University of Bath, 10 West 3.25, BA2 7AY, Bath, UK

**Keywords:** Co-production, Obsessive compulsive disorder, OCD, Blended intervention, Relapse prevention intervention, Patient and public involvement, PPI, Person Based Approach, Expert By Lived Experience

## Abstract

**Introduction:**

Co-production involves researchers, practitioners and people with lived experience working in a collaborative manner, with shared power. The potential benefits of co-production are well documented. However, there is little research describing the experience of having been involved in co-production from the perspective of Experts By Lived Experience (EBLE). The aim of the present study is to explore the experiences of EBLE of obsessive-compulsive disorder (OCD) on their involvement in co-producing a blended intervention to prevent relapse for OCD.

**Methods:**

Five EBLE took part in semi-structured interviews enquiring about their experiences of co-producing a relapse prevention intervention. Reflexive thematic analysis was used to analyse the data.

**Results:**

Four themes were developed: (1) Welcome but unexpected therapeutic benefits; (2) The parameters of a safe space; (3) Genuine co-production brings meaningful change; and (4) Navigating the challenging terrain of co-production.

**Conclusions:**

Overall, EBLE reported their involvement in the co-production process to have had positive impacts on both the development of the intervention and their own personal recovery journey. EBLE valued the safety created within the group, and the importance this had for allowing them to speak open and honestly about their experiences and the difficulties that can arise with the nature of the work.

**Supplementary Information:**

The online version contains supplementary material available at 10.1186/s12888-024-06355-1.

## Introduction

Obsessive-Compulsive Disorder (OCD) affects 1–3% of the population (Ruscio et al. 2010) and carries a high clinical and humanistic burden (Kocher et al., 2023). Cognitive Behavioural Therapy (including Exposure and Response Prevention) (CBT) is the first line psychological intervention for OCD (National Institute for Health and Care Excellence [NICE], 2005, 2013), and its short-term efficacy is well established (Öst et al. 2015). However, post CBT, up to 60% of patients experience relapse (Eisen et al. 2010; Marcks et al. 2011; O’Neill and Feusner 2015) with 85% of relapses occurring within the first 12-months (Braga et al. 2010). Despite this, relapse prevention (RP) is not a part of standard care and research addressing this gap is scarce. To date, only two published studies have investigating RP for OCD: the first is a randomised controlled trial of four 90-minute face-to-face sessions and 12 telephone consultations (*n* = 8) compared to an active control (*n* = 12). At six-month follow-up, 75% of the intervention group (*n* = 8) had maintained their gains, in comparison to 33% of the control group (*n* = 10) (Hiss et al.,^1994^). The second published study is a case report (*N* = 1) of a stand-alone mobile application (used over a 2-week period), which was reported to have assisted in maintaining and increasing therapeutic gains, at 2-year follow up (Pascual-Vera et al. 2018). Despite the small sample sizes, both studies provide promise for the potential utility of RP interventions for OCD, however, further contemporary robust exploration is required. Namely, research focused on understanding the needs of the target group within the context of OCD recovery. Such research should be conducted prior to, or in parallel with, any further development of interventions aimed to prevent relapse.

Historically, the inclusion of Patient and public involvement (PPI) in the development and evaluation of mental health interventions has been lacking, absent, or at worst tokenistic. This is problematic on numerous fronts. Without meaningful PPI involvement and incorporation of PPI perspectives, the acceptability of the intervention will likely be at risk (Bucci et al. 2019). Contemporary approaches such as the Person Based Approach (PBA; Yardley et al. 2015) to intervention development aim to address this issue by placing PPI at the heart of the research process. This means Experts By Lived Experience (EBLE) are actively involved from research inception to completion. This approach aligns with the practices of co-production, which involves researchers, practitioners and the public working together, with the intention of sharing power and responsibility throughout the entirety of the research project (Farr et al. 2020). The underlying assumption is that complex problems cannot be solved via scientific expertise alone. To reach a solution that is relevant, effective, and accessible to those affected, it is imperative to involve those with ‘expertise by experience’ of the problem (Polk, 2015; Turnhout et al. 2020). A recent systematic review on the co-production of digital mental health interventions supports this premise, finding that the use of co-production led to more culturally sensitive and acceptable digital mental health interventions (Brotherdale et al. 2024).

Theoretically, co-production provides a transformative way of research being conducted collaboratively with those personally affected, shifting the emphasis away from research that is ‘done to, for, or about’ a particular population (Dewa et al. 2021). However, careful thought and attention must be given to *how* the principles of ‘co-production’ (outlined in guidance, e.g., National Institute for Health Research, 2024) are *actively* incorporated within the research process (i.e., how power is redistributed so that it is shared, how the perspectives and skills of all involved are included, how relationships are built and maintained, etc.). Failing to do so can instead perpetuate the dynamic of ‘the researcher’ and ‘the researched’ rather than disrupting this notion (Lambert and Carr 2018). In addition, the intended benefits of co-producing the research will likely be lost, and, at worst, unintended harm could be caused to those involved.

To the best of the authors knowledge, to date, there are less than a handful of published studies that report on utilising co-production methods with EBLE of OCD within or throughout the research process. Of the studies that do, positive benefits to the outcomes of the research are reported. For example; Waite et al. (2023) engaged five adolescences with lived experience of OCD and two parents to review an existing CBT treatment for adolescent OCD. The EBLE participated in interviews and provided written feedback. This involvement led to revisions of the intervention workbooks and materials, improving both the acceptability and usability of the intervention. Further, a qualitative study that aimed to understand the support needs of parents of children with OCD, described consulting with EBLE in the development phases of the study and involving an EBLE as a co-researcher who was trained in qualitative methods (Sowden et al. 2023). Sowden et al. (2023) reflected on how the use of co-production had enhanced the sensitivity and quality their work as well as the credibility of their findings. However, no published studies were identified that included in-depth reflections on other aspects of the co-production process within the research cycle. Further, no published studies have aimed to understand the experience of co-producing research from the perspective of the EBLE with OCD.

The current study is nested within a wider research project, which co-produced a blended CBT intervention aimed to prevent relapse in OCD. The wider research project employed the PBA (Yardley et al. 2015) to intervention development, involving EBLE contributors at all stages of the research process (i.e., identification of need; co-creation of potential solution; grant funding application; co-production of content for intervention; evaluation of created intervention). The focus of the current study is on the experience of the EBLE^1^ contributors who formed a steering group (SG), to co-design and co-produce the content of the blended intervention. The blended intervention comprises: (1) 10 small interactive group sessions facilitated by a qualified therapist via Microsoft Teams, with each session focusing on a distinct theme implicated in OCD relapse; and (2) a corresponding mHealth app, which provides an in-depth evidence-based toolkit of resources that enables users to put theory and strategies discussed in the group sessions into practice. This content was co-produced in collaboration with two qualified clinical psychologist researchers (CPR) with expertise in the treatment of OCD. Thus, the aim of the current study is to gain an in-depth understanding of the experience of being involved in the co-production of the above-mentioned blended intervention from the perspective of the EBLE contributors.

## Methods

### Forming of steering group (SG)

The idea to design and develop a relapse prevention intervention for OCD was co-created with nine EBLE over several years via conversations and discussions between JM and EBLE. Six of the nine EBLE initially became known to JM when they met via their attendance at OCD charity conferences which are predominantly attended by people experiencing OCD and their loved ones. JM initially met two of the nine EBLE when working clinically in her role as a clinical psychologist. JM reconnected with these two EBLE individually, approximately five and six years later via an OCD focused network and an OCD conference. Finally, JM initially met one of the nine EBLE through her academic role where they work collaboratively on a clinical psychology training programme. Discussions between JM and EBLE focused on the shared concerns and dissatisfaction with the absence of support available post-CBT for OCD and the negative impact this has on the individual and the wider system. Many EBLE had ideas regarding what is needed and what a RP intervention could comprise. A grant application was written by JM incorporating the ideas from the shared discussions. JM contacted each of the nine EBLE via email to advise of the plan to apply for grant funding to take the idea forward. JM invited the EBLE to provide letters of support to accompany the grant application.

The nine EBLE mentioned above were invited via email to be involved in the research in the capacity of a EBLE contributor and to form part of a steering group (SG). The email invitation included an information sheet outlining what co-production is, what taking part in the process of co-production would involve, details of the time commitment, dates of the SG meetings and details of payment for EBLE’s time. If EBLE were interested in finding out more and potentially being involved, EW (CPR) arranged a time to meet with the EBLE for an eligibility call via telephone or video call. To join the SG, EBLE were required to be: aged *≥* 18 years; have undertaken a course of CBT for OCD which had enabled them to make therapeutic gains; experienced a relapse in OCD post-CBT intervention; be able to attend *≥* six of the scheduled SG meetings and be fluent in English. EBLE needed to be at a point in their recovery where they felt able to manage being in a group and to engage in open discussions about OCD and relapse. These requirements were discussed individually with each EBLE during the eligibility call.

Prior to conducting the eligibility calls, EW consulted with the lead of an OCD charity (led by EBLE of OCD) who had experience in the effective running and management of both focus and working groups involving people with lived experience of OCD. EW sought guidance from the charity lead regarding the process of engagement and setting up of the SG. This guidance was incorporated into EW's approach to the eligibility calls. For example, during the eligibility calls EW invited and encouraged each EBLE to ask any questions or express any concerns they had, to aid the EBLE to determine if taking part in the SG would be a good fit for them and if it was the right time in their own recovery journey to be involved. In addition, EW sought the views of each EBLE regarding what they perceived would be important for the CPR’s to consider during the SG meetings to ensure psychological safety was established and maintained within the group. The aspects reported by EBLE were collated by EW and shared (anonymously) with the SG members in the first meeting. These aspects contributed to forming the foundation of the SG’s shared principles for engagement. Following the eligibility call the EBLE was given time to decide if they wished to be involved and join the SG. If they did, they were asked to provide their informed consent to be involved. Of the nine EBLE invited, five decided to take part and a further EBLE was identified through snowballing methods. No participants were excluded based on the inclusion criteria. All EBLE provided their informed consent to collaborate on the research as a EBLE contributor SG member.

### Method of SG meetings

Over a period of 18-weeks, the SG members and CPRs (JM & EW) met virtually via Microsoft Teams on 14 occasions. Meeting duration was two-hours per meeting with a 15-minute break. This allowed time for introductions and wrapping up, which was included to cultivate and perpetuate a safe, friendly and welcoming environment. The first SG meeting was dedicated to the collaborative fostering of the SG culture. The intention was to create a culture of respect and trust from the outset, a non-judgmental environment where all SG members would feel empowered to express themselves without fear of criticism or stigma. To facilitate this, the aspects relevant to psychological safety as previously identified by EBLE in their eligibility calls (via the process outlined above) were shared with the group and further discussed. EBLE contributors were invited to contribute during the SG meetings in ways that they felt most comfortable (e.g., via the chat function or to turn their camera off if/ when needed). The facilitators (JM & EW) aimed to normalise these different ways of contributing by modelling such during the SG meetings.

The facilitators emphasised how important it was to them that the intervention was genuinely co-produced. They shared that this was their first experience of co-producing an intervention, and thus, they would also be learning throughout and welcomed all feedback. The facilitators named processes relevant to the principles of co-production and invited the EBLE’s contributors to think with them and share ideas for how these principles could be collaboratively embodied. The facilitators shared their preliminary ideas on the same (e.g., to support the sharing of power the facilitators wished to invite EBLE SG members to chair future SG meetings). It was made clear that the intention was to review these processes over the progression of the meetings to assess what was or wasn’t working well, and new ideas were welcome at any stage. Following this, EBLE SG members were invited to share in the co-setting of the SG’s ground rules. Facilitators made it clear that the safety and wellbeing of the EBLE contributors was of primary importance. The proposed agenda for each meeting was sent in advance and EBLE contributors were invited to contact the facilitators ahead of time if they had concerns. At the beginning of each meeting all SG members entered a rating of their mood (0–10) in the chat. The facilitators made themselves available for 30 min after each SG meeting. EBLE contributors were reminded at the beginning and end of each meeting that they were welcome to seek support from the facilitators during this 30-minute time slot if anything difficult had come up for them.

During the remainder of the SG meetings, one distinct theme implicated in OCD relapse was discussed at each meeting. In the first part of the SG meeting, EBLE contributors reflected on if/how the theme being discussed had been relevant to their personal recovery journey. Following this EBLE contributors shared their thoughts and reflections. The remainder of the meeting time was dedicated to the EBLE contributors’ generation of ideas for how the theme could be addressed in both the small group sessions and corresponding mHealth application. When creating the content for both parts of the blended intervention, EBLE contributors drew on their own experiences of what they had found helpful/ unhelpful. In addition, they discussed ideas pertaining to what they perceived would have been useful to their recovery, if it had been available at the time. The EBLE contributors developed ideas for a range of activities including self-guided interactive personal challenges and tasks to be included in the intervention. Many of such were designed to be embedded within the mHealth application component of the intervention. EBLE contributors deemed it important for the mHealth application based tasks to be designed to be used in-between (and beyond) the group sessions. EBLE contributors envisioned the mHealth application akin to a *“therapist in my pocket”* being available to the user to access and make use of at any time when needed. The SG’s shared vision was for the intervention to empower the individual user to become their *“own therapist”* taking charge of their own recovery journey. In addition, EBLE made short videos to explain various aspects of the intervention or to provide examples of how undertaking a particular activity had assisted their recovery. The facilitators drew on their theoretical knowledge while working with the EBLE contributors to ensure all content was theoretically informed. In parallel, 10 Computer Science Master students at the University of Bath were engaged to develop mock prototypes for the mHealth application component of the blended intervention, incorporating the ideas developed during the SG meetings. One Masters student was invited to attend the 7th SG meeting and presented a mock working prototype of the mHealth application to the SG members. The aim of this was to gain EBLE contributors initial feedback on how the content they were generating was being captured and realised. All EBLE contributor SG members (*N* = 6) remained involved until completion of the project.

### Evaluation of the SG experience

The need for an evaluation of the intervention development process was suggested by a EBLE contributor during the first SG meeting. All SG members were in favour of an evaluation. It was agreed that the evaluation study design would be co-produced. In the penultimate SG meeting, a qualitative interview study aiming to explore EBLE contributor’s experiences was planned. It was decided that interviews should be conducted by a researcher(s) external to the project, who had not been involved in the intervention development phase. It was also agreed that the CPRs (JM & EW) ) would only access the interview data once it had been fully anonymised. The lead CPR (JM) agreed to seek and appoint researcher(s) (with no previous involvement in the project) to conduct the interviews and analyse the qualitative data collected. An email invitation inviting expressions of interest for the role, from researchers with experience of conducting qualitative research was sent to all PhD students in the Department of Psychology at the University of Bath . Two researchers responded to the email and JM met with each individually to discuss their experience of conducting qualitative interviews and analysis. Both researchers had previous experience of conducting interviews with participants who had experience of mental health difficulties. Both researchers also had experience of conducting qualitative research from inception to publication. One researcher had personal experience of OCD. In discussion with the researchers, it was decided that their experiences were complimentary and thus they would undertake the interviews and data analysis collaboratively.

### Current study

EBLE contributors of the SG were contacted by the researchers (NH-S & TJ) via email to invite them to participate in an interview study aimed to understand their experiences of being involved in co-production. Five of the six EBLE contributor SG members consented to take part, aged between 34 and 46 years (*M* = 38.75, *SD* = 5.85). All participants identified as White British and three as female.

The principles of information power (Malterud et al. 2016) were employed to determine if the data obtained from the sample of five participants was sufficient for achieving the aim of the current study. This was done by evaluating the nature of the study aim, the specificity of the sample recruited, the quality of the interview dialogue obtained and the proposed strategy for data analysis. The study aim is relatively narrow and the experiences of the sample are highly specific and relevant, with variation in responses indicating diversity in participant perspectives. The quality of the interview dialogue was strong with richness and participants providing deep and insightful information. The combination of these factors suggested that that data gathered from the five participants was sufficiently robust and varied enough to provide meaningful insights. In addition, given the lack of existing research in this area, this study provides an initial exploratory investigation with sufficient information power to generate foundational insights.

### Data collection

Ethical approval was gained from the University of Bath Psychology Research Ethics Committee (21–214). Prior to submission of the ethics application for the current study, the CPR’s (JM & EW) in consultation with an EBLE (not involved in the SG) developed preliminary questions and areas of focus for the semi-structured interview. The interview schedule (Appendix A) comprised four sections of focus: (1) Group processes and psychological safety; (2) Power dynamics and co-production methodology; (3) The experience of working with others impacted by OCD; and (4) Future research recommendations. The final question of the interview asked participants their views regarding whether they believed there were any potential benefits to creating an intervention via co-production. The researchers (NH-S & TJ) who conducted the interviews discussed the semi-structured interview schedule. They built on the interview questions, adding prompts ahead of commencing the interviews and throughout the interviewing process. The researchers (NH-S & TJ) had no prior involvement in or contact with the EBLE contributor SG members. The intention was that due to the researchers having no prior involvement, it would allow the EBLE contributors participating in the interview to be more forthcoming in their evaluations of their experience. For the same reason, NH-S and TJ led the data analysis.

Written informed consent was gained prior to conducting and recording the interviews. The Interviews were conducted via Microsoft Teams between September and November 2022. Interviews ranged in duration from 38 to 61 min. Interviews were recorded and transcribed using the transcription function on Microsoft Teams. Transcripts were checked and corrected by (SM, AB & YZ). Participants received £20 following the interview as a thank you for their time and expertise.

### Data analysis

Reflexive Thematic Analysis (Braun and Clarke 2006, 2021a) was used to analyse the data. This method entails identifying, analysing, and reporting patterns of meaning within a qualitative dataset. An inductive, ‘data-driven’ approach was taken, meaning that the analysis stuck closely to the data and was not driven by a pre-existing theoretical framework. An experiential perspective guided the analysis, with participants’ experiences, perspectives and interpretations prioritised throughout. This was underpinned by an essentialist/realist epistemological framework. Semantic codes reflected explicit meanings of the data and a knowable reality (Braun and Clarke 2013).

Throughout the study, the researchers actively considered how their prior knowledge, experiences, identities, and beliefs may have influenced the research. This included lived experience of OCD (N-HS), receiving (NH-S) and delivering CBT for OCD (JM & EW), conducting research with EBLE (JM & EW), and previous roles within mental health services (JM, EW, NH-S & TJ). Reflexivity was achieved through regular team meetings where the authors discussed how they individually made sense of the data.

Data was analysed following Braun and Clarke’s (2006; Braun and Clarke 2021a) six-phase process. In phase one, NH-S and TJ both read and re-read all transcripts individually to familiarise themselves with the data. In phase two, NH-S and TJ began by coding the first interview individually, before meeting to discuss their different understandings and interpretations. The remaining four interviews were then divided equally, with each researcher conducting individual line-by-line coding for their assigned interviews. In phase 3, NH-S and TJ met to consolidate codes and look for overlap. Following this, they collaborated to generate initial themes and subthemes. In phases 4, 5 and 6, themes and subthemes were reviewed, defined, and consolidated throughout the process of writing, with input from JM and EW.

## Results

Four themes were developed, namely: (1) Welcome but unexpected therapeutic benefits, which comprises three subthemes; (2) The parameters of a safe space; (3) Genuine co-production brings meaningful change; and (4) Navigating the challenging terrain of co-production, which comprises three subthemes.

### Theme 1: Welcome but unexpected therapeutic benefits

This theme reflects the unexpected secondary benefits participants reported deriving from taking part in the co-production process. Across the dataset, participants described how being able to share their personal experiences led to additional opportunities to learn from and help others, which were experienced as therapeutic. These unexpected therapeutic benefits are explored across three subthemes.

#### Not the only one: the power of shared experience

This subtheme reflects the sense of validation and power that participants experienced from being able to engage and collaborate with other EBLE of OCD. Prior to the SG, some participants had felt misunderstood by others and experienced social isolation due to their OCD. The opportunity to be around others with shared experiences of OCD who understood the realities of the disorder led to a sense of connection, shared acceptance, and universality, which was of therapeutic benefit. Participants reported the sense of validation came from others in the group with lived experience, and also from the facilitators.

P5 described how they valued being around others who *“got”* the experience of OCD, meaning that they did not have to explain themself:

*Overall it was*,* I think quite*,* um*,* validating really*,* to hear people who just understood and got where you were coming from. Because sometimes other people don’t even have the language to be able to have that conversation with you and certainly not the experience where you can actually talk about OCD in any meaningful way with people*,* because they just don’t understand it.*

P3 explained how they experienced the group as comforting. Key to this was meeting others with OCD for the first time, which helped to normalise their experience and provide a sense of support.

*​​It’s horrible to know that others are going through the same sort of difficulties and challenges*,* but there’s also a sort of comfort in knowing you are not alone and you are not the only one that’s kind of experienced that … I guess it’s sort of normalising the fact that I’m not the weird freaky person that I feel like I am.*

#### Embracing new perspectives

This subtheme highlights the process of learning from what others shared in the SG, pertaining to the difficulties of overcoming OCD and the strategies that others had employed in their recovery journeys. Across the data, participants reported that they had taken something away from the experience that would positively influence their own recovery. These learnings and new perspectives were seen as invaluable, with P3 describing how they had developed self-compassion for themself from empathising with others in the SG:

*​​It definitely helped me be more compassionate towards myself and it gave me that perspective that I wasn’t the only one to have not coped*,* or [to] have been overwhelmed*,* or massively impacted*,* or for it to have ruled my life to the extent that it did.*

Participants reported that after interacting with the SG they had felt more motivated to engage with strategies they had previously used/learnt in therapy. Participants reported feeling inspired by how others responded to setbacks, appreciating the realities of people’s lives, and seeing how people were able to flourish in spite of their OCD.

*I think it actually had a positive impact on our own OCD actually*,* and I think you know*,* a lot of us said actually in [SG] sessions*,* we went away.… and we felt you know inspired to face an exposure task*,* or*,* you know*,* cut down on a compulsion or just like try a little bit harder*,* kind of thing*,* with the OCD and it just gave us that little bit of energy. Yeah*,* so I think it had like*,* I think it- it kind of genuinely helped our own conditions* (P4).

EBLE SG members shared a range practical tips, strategies and resources to be incorporated into the intervention. However, this also allowed SG members to learn from each other during the meetings. Such learning was reported to have continued once the SG meetings had ceased; *“Books […] or websites*,* or blogs*,* or whatever […] we shared a lot of resources we found helpful”* (P3). This meant participants were able to engage with new ideas from direct participation in the SG, alongside contributing to the intervention development.

*When you heard how different people dealt with moments of difficulty*,* uh and I think nearly all of us in the group seemed to bounce off and then a few weeks later you’d get ‘I did the thing that you mentioned*,* and I found that really helpful’* (P2).

#### Empowerment in paying it forward

This subtheme reflects the unexpected sense of empowerment that participants experienced from being involved in co-producing an intervention designed to help others in the future. Participants described being motivated to take part in the SG because of a drive to help others and saw co-developing the intervention as a way to prevent others from having similarly negative experiences. However, participants reflected that they had not anticipated the positive sense of momentum and spirit that undertaking this work could bring.

*Meeting others through the group who’ve also had that experience kind of only increase[d] my passion*,* to I guess*,* try and help others through our not so great experiences*,* to try and make sure that somebody else might have a slightly – not easier*,* but more kind of clear pathway out* (P3).

The knock-on effect of this was a sense of empowerment. Participants described feeling a sense of purpose and pride in being able to contribute to the development of the intervention. Participants were glad to make a positive contribution to the OCD community through both normalising the experience of lapses/relapse, the potential to change the narrative regarding misconceptions about recovery and developing an intervention designed to help others maintain gains and keep well.

*A lot of people underestimate how many times people can relapse […] I’ve always felt in the past that the treatment was almost*,* “right*,* this is the treatment*,* if you do this*,* you’ll improve’ and then there’s no discussion after that about what happens” […] [the RP intervention is] a real positive for the OCD community to-to normalise lapses and relapses because we-we tend to be so hard on ourselves* (P5).

Participants also found this to have a cathartic effect, by providing them with an opportunity to turn something negative into a positive. *“I found it incredibly empowering […] it allowed me to kind of put my suffering to good use to help other people”* (P1).

### Theme 2: The parameters of a safe space

This theme focuses on the factors that participants reported as being integral to the success of the SG and their ability to engage fully in the co-production of the intervention. Across the dataset, participants reported that a friendly, caring, and compassionate environment had been created within the SG from the beginning, which was embodied by each of the SG members, including the facilitators. Participants reported the value of being invited to set ground rules, and described appreciating that the facilitators had made it clear that they would be available to support everyone. Participants reported that the attention given to their welfare had been essential for enabling a sense of safety and containment within the SG, which was reported to be a vital part of the group dynamic.

*We always knew that we had people to talk to*,* that we could always contact [first facilitator] and [second facilitator] […] it was made clear always that the priority was our happiness and our safety* (P1).

Participants reported that a non-judgmental attitude was present throughout the SG meetings by all members of the SG. Participants described that they felt safe and able to be open and vulnerable within the SG. This helped participants express themselves more fully, leading to the development of deep connections with others and richer, more productive discussions.

*When I was in the group*,* even then talking about some very personal and challenging experiences it*,* is very difficult*,* but they did make me feel very comfortable um to do that. It did feel like a safe space* (P5).

Participants reported that they valued the collaborative nature of the project, which was underpinned by respect for everyone’s experiences. Participants recalled previous experiences of not being well-listened to by mental health professionals, but felt they were treated as equals in the SG. Participants emphasised that *“every voice was heard”* (P4), and that the expertise of the EBLE was valued at least as highly as professional expertise. This appeared to be a fundamental point for the success of the co-production method.

*It did really feel collaborative – like they valued what we had to say just as much or even more than their own kind of professional knowledge or experience which doesn’t happen that often I’d say* (P3).

Participants reported that they were kept well informed about the structure of the sessions, particularly if they would be facing anything difficult or potentially triggering. This helped participants to feel more involved and in control of their participation. Participants expressed how they valued this open, non-pressurised environment, and that there was never an obligation to share if they did not want to.

*I can’t see a way that they [facilitators] could better it*,* they clearly gave it a lot of thought. The structure*,* the layout*,* the willingness to change*,* erm the lack of pressure*,* um keeping people in the know*,* they walked a very fine line*,* you know*,* it’s important to keep people aware of what’s going on and up to date*,* but then to keep the pressure low* (P1).

Participants remarked on the friendly atmosphere of the group, and often referenced the bonds and friendships that were developed, culminating in a sense of belonging and a lasting sense of community. P1 said it was a *“real pleasure”* to spend time with others in the group, and that they were a *“team”.* P3 spoke of the *“strength of bond and trust”* within the group, underpinned by a *“shared understanding”* from their experiences. *“It felt like a really*,* kind of*,* cohesive*,* strong*,* light support group that I just had not anticipated in any way […] we formed quite a – quite a strong bond”* (P3).

### Theme 3: Genuine co-production brings meaningful change

This theme captures participants’ perspectives on the prospect of being involved in co-producing research and how this changed over the course of their involvement in the SG. Participants described that, at the outset of the SG, they felt a mixture of hopefulness and hesitancy at the prospect of ‘co-production’, which was provoked by previous negative experiences. Thus, experiencing the SG as a genuine attempt at co-production was reported to be surprising but encouraging: *“Very often as [someone with personal experience of OCD] you feel that people contact you for your information and they kind of run away and do stuff with it*,* you know?”* (P1).

*I was a little bit (pause) apprehensive because […] with co-production*,* sometimes it can be a little bit tokenistic*,* or*,* you know*,* it kind of looks good on paper*,* so I was a little bit worried*,* you know*,* are we genuinely going to have our voices heard […] I was pleasantly surprised that it is actually a methodology that can work when it’s done properly”* (P5).

At the heart of participants’ experiences of the SG was the ability to bring about change through co-production. Participation in the process led to positive feelings towards co-production as a methodology, and participants were keen to see it further utilised in the field. Further, participants perceived there to be a limit to what could be gained in research without lived experience input, positioning it as a vital element in developing successful treatments. This was seen as particularly important in the context of a stigmatised and *“such a misunderstood disorder”* (P5) like OCD: *“You can only theorise so much from what you ‘think’ you know*,* but you gotta [think] is that right? Are we on the right track here? Because if we’re not*,* it’s a big waste of time”* (P2).

*I think it’s really easy for professionals to […] think they understand the experience (pause) but they are actually getting that from maybe like textbooks or from other professionals and I think you would miss (pause) some of the nuances of what makes treatment either acceptable or difficult*,* what makes people stay in treatment*,* what makes them drop out* (P3).

Collaboration between researchers, clinicians and EBLE was viewed as a fundamental part of this endeavour. It was understood that each individual could provide a different perspective. The outcome of this combined working was the hope that it would lead to a more holistic understanding of OCD within the context of relapse and to the shared aspiration of an effective intervention.

*By putting our heads together*,* both the person who’s walking through that journey and the person who’s walking through it with you*,* it feels as though you then should get a 360 view of how to make treatment as tolerable as it can be and acceptable so people don’t drop out* (P3).

In the end, participants expressed being excited about the blended intervention and the eventuation of the mHealth application they had co-produced. Participants emphasised how their lived experience input gave it *“that personalised edge”* due to the fact that *“we can relate to what you’re going through”* (P3). Participants had high hopes for both the intervention itself, and for what its potential success could mean for the popularity of co-production in research: *“I’d like to think that*,* if a good product or service comes out of it*,* you know*,* it might encourage more co-production in the future”* (P5).

### Theme 4: Navigating the challenging terrain of co-production

This theme reflects the challenges of taking part in co-production, as discussed by participants. Many of the highlighted challenges seemed inherent to the territory of co-production, alongside participating in a group to undertake this work. This theme comprises three subthemes, with each depicting a different challenge some of which were unavoidable, and others which could be overcome.

#### Group dynamics

This subtheme reflects the difficulties that participants reported experiencing that stemmed from the nature of group working, which at times led to unhelpful comparisons. Despite the positives of working as a group towards a shared goal, participants acknowledged some of the difficulties; as P5 succinctly put it, *“Well*,* it’s group dynamics. Just normal*,* isn’t it?”.* One of the challenges is highlighted by the dissonance between P2 and P4’s accounts of participating in the SG:

*Sometimes I personally might have found it a little bit hard to get my voice heard […] there was maybe some characters who spoke up a bit more and*,* you know*,* and naturally in that-in that situation you’ve got sort of a bit of a dynamic between the kind of louder voices and maybe the kind of slightly quieter voices* (P4).

*I never felt anyone was dominating the conversation*,* probably other than me a little bit and um yeah*,* I-I think it felt (laughs) evenly balanced* (P2).

Not all group members felt like participation was always equal. Whilst some felt confident in sharing their opinion and being heard, others described struggling to compete with those who were quicker to contribute or were more outspoken. Some participants expressed feeling concerned that they may not have contributed enough to the group in comparison to others: *“Everyone had sort of said what I was thinking and I had nothing more to add and I don’t know sometimes I worried if I was*,* you know*,* bringing enough positive input to the group”* (P5).

This challenge of comparison extended to discussions about recovery. Whilst these discussions were often experienced as encouraging, at times participants reported that they had negatively compared themselves and their progress to others, which could be disheartening.

*Initially I felt quite self-conscious that maybe I wasn’t as either as far on in my recovery*,* or that I wasn’t fighting as hard as other people*,* and so I think in the very first few initial groups – I felt a little bit like (pause) I should be doing more – I should be fighting for it more* (P3).

#### Participation in the context of OCD

This subtheme reflects the interplay between participants’ willingness to be vulnerable and share within the SG for the benefit of others, and the balance of managing their own wellbeing within this context. Although participants were eager to drawn on and discuss their lived experience as a part of the co-production process, they acknowledged that recounting their experiences, and hearing about the experiences of others, could at times be triggering. For some participants, participation in the interview for the current study, also evoked difficult feelings:

*Because of the […] trauma that it’s caused me over the years*,* I still find it quite difficult. Like even*,* like a meeting like this can be a little bit difficult for-for me […] I guess it makes me feel quite vulnerable*,* talk-talking about it* (P5).

For some participants, this evocation of such feelings was surprising. However, for others, it was described as being expected and understood to be part and parcel of working in a group on a topic that was personally significant.

*I knew that it was gonna be hard work and I knew that a load of OCD people together or people with OCD together*,* things would come up that I wasn’t quite ready for or would potentially be upsetting […] some of the conversations were hard* (P1).

Participants emphasised that this risk was not a reason to avoid co-production. Instead, it highlighted the importance of facilitators having a good understanding of the condition being explored, implementing safeguarding practices, and ensuring EBLE needs are put first.

*You’re all equal and you don’t want special*,* erm you know*,* kind of attention as it were*,* but […] they [facilitators] need to be considerate to the fact that you do have these individual needs because of your condition* (P1).

#### Logistical considerations

This final subtheme reflects the logistical elements of participants’ experiences and includes factors which facilitated and hindered their ability to engage with the SG. Hosting meetings online meant a wider variety of people from different geographical locations could attend, but the virtual environment was reported to impede engagement for some: *“I think it was just sort of little*,* little*,* extra comments that you kind of lose slightly being on [Microsoft] Teams and Zoom when you’re not in that situation where you’re in person”* (P4).

For others, having the group take place online was experienced as a facilitator of engagement, as it mitigated some of the difficult group dynamics discussed in 4.1, e.g., different levels of participation.

*The chat function I liked as well because quite often lots of us would be having similar thoughts so we could write in that […] sometimes if I felt a bit unsure about saying something*,* um*,* and having someone else like agree with it or ‘clap’ or ‘thumbs up’ or put something in the comment was quite – I guess quite validating and reassuring in a way* (P3).

But there were some barriers that could not be as easily solved, such as real-world commitments and time-limited sessions. Flexibility from facilitators, both in terms of understanding when sessions could not be attended by some SG members and allowing participants to share their opinions via multiple avenues, was reported as important by participants: *“Quite a lot of us are parents*,* some of us missed meetings*,* and I’m sure at times that would be a pain for them [facilitators] […] you waste the continuation”* (P1).

## Discussion

This study aimed to gain an in-depth understanding of the experiences of EBLE contributors involved in co-producing a blended RP intervention for OCD. This is the first study to explore the experience of co-production from the perspective of people with lived experience of OCD. Four themes were developed through the use of reflexive thematic analysis. The first (Welcome but unexpected therapeutic benefits) comprised three subthemes that reported on the various benefits participants described unexpectedly experiencing as a result of engaging in the co-production process. Participants perceived these benefits to have a therapeutic element that had positively impacted them. The second theme (The parameters of a safe space) focused on the elements participants reported as being vital to the success of the SG, which aligned with the importance of establishing psychological safety. The third theme (Genuine co-production brings meaningful change) revealed the initial hesitation participants held due to previous negative experiences of research co-production involvement. This was contrasted by participant’s reports of experiencing a genuine attempt at co-production via their involvement in the SG. The fourth and final theme (Navigating the challenging terrain of co-production) comprised three subthemes that explored the challenges inherent to co-production and group working, including difficulties with group dynamics, vulnerabilities with sharing their lived experience, and logistical constraints.

Although co-production is not intended to provide any therapeutic impact, all participants reported benefits such as connection, validation, universality, catharsis, and empowerment, which were experienced as such (Theme 1). These unexpected gains can be seen as a welcome companion to the co-production methodology and are consistent with reports from the wider PPI literature (Richmond et al. 2023). The personal agency gained from making meaningful contributions helped participants to feel purposeful and valued. This finding aligns with previous qualitative research on young people’s experiences of co-production (Mayer and McKenzie 2017), where participants reported greater self-esteem and self-efficacy from their involvement. Sharing experiences of OCD allowed participants to see they are not alone in their experiences, giving rise to a sense of universality, a key process in therapeutic groups (Yalom 1995). Furthermore, participants in the current study were able to gain new perspectives through their SG participation, leading to enhanced self-compassion, the identification of new self-management strategies, and renewed motivation to engage in tackling and keeping on top of their own OCD. Participants emphasised how validation and normalisation that transpired through shared understanding had been instrumental in facilitating open and honest discussions. The element of peer support provided by the SG appeared key to this effect. The impact of shared experience in the context of mental health is often discussed in the peer support literature as a core component of what makes peer-delivered services effective (Stefancic et al. 2019). Whilst peer support services and co-production are different entities, the power of shared experience seems core to both, and should be taken into consideration when implementing co-production within research.

The friendly, supportive and contained environment of the group was a key facet to its success (Theme 2). Participants were complimentary about the other members of the SG. Participants also remarked on the practices implemented by the facilitators to ensure the safety of the group, such as collaborative boundary-setting, clear communication, and flexibility. Bell et al. (2023) highlight the importance of psychological safety within co-production, stating that participants should feel they can speak up without fear of stigma or judgement. Participants in the present study remarked on how comfortable they felt around one another, and that they could contribute as much or as little as they wanted. Bell and colleagues (2023) emphasise the importance of the facilitator role in maintaining group dynamics within co-production. This was echoed by the current participants, with feedback that sessions were well managed, with facilitators striking a delicate balance between not commandeering the sessions, but also providing space for every voice to be heard.

Participants described initial mixed feelings of hope and apprehension towards their involvement in the SG (Theme 3). While their rapport and trust in the facilitators had motivated them to take part, previous negative experiences with different research, had laid the foundation for concern about their involvement potentially being tokenistic. Tokenism refers to the practice of doing something as a symbolic gesture that gives the appearance of equality and inclusivity and is a wider concern throughout PPI (Ocloo and Matthews 2016) and co-production research (Farr 2018). Although equality and reciprocity are key tenets underpinning co-production, theory does not always translate into practice, particularly when power dynamics (and other elements integral to co-production) remain unaddressed (Turnhout et al. 2020). In the current study, facilitators worked to address power through clear and open communication, payment to recognise the expertise and value of EBLE’s contributions, invitations to actively share in ‘power’ (e.g., EBLE contributors were invited to share in the chairing of the SG meetings), and co-design of the aim, method and procedure of the current study aimed to evaluate EBLE contributors experiences of co-production. These strategies resulted in participants experiencing the group as an equal space. For co-production to be successful, it is important for researchers to consider that EBLE may have had previous negative experiences, and that addressing issues relating to power dynamics and collaboration will need to be done at the outset and throughout to ensure that this is not perpetuated. Whilst the efforts of individual researcher(s) and clinician(s) are important and essential, their capacity to influence wider change should be considered within the context of the system in which research is conducted. Researchers/ clinicians are operating within the realities of a system where inherent power dynamics exist that cannot be easily escaped (e.g., tertiary education or health care settings, employer/employee, researcher/researched). Without systemic change at an institutional level, issues relevant to power will continue to be perpetuated. Institutions need to lead on the embedding of such practices and hold space for meaningful dialogue that addresses the broader issues around who holds the power within research. Further endeavours (which are beyond the scope of the current study) to reduce power imbalances by challenging existing societal structures need to be carefully considered and undertaken.

In light of their positive experiences, participants were strong advocates of co-production as a methodology and emphasised the importance of lived experience input in mental health research. Without lived experience perspectives, participants felt that only so much insight could be afforded, and that nuances regarding the lived reality of OCD would be lost. Previous research has highlighted the various two-way benefits of co-production, with the integration of lived experience input leading to improved research quality, greater impact, more holistic understandings, and a sense of inclusion and empowerment (Oliver et al. 2019). This may be particularly important in the context of mental health disorders like OCD, which can be heavily stigmatised and misunderstood (Homonoff and Sciutto 2019; Ponzini and Steinman 2022).

However, as with all research, the benefits must be weighed against the potential costs. Participants discussed the challenges of co-production, specifically group dynamics, vulnerability, and logistics (Theme 4). Vulnerability was discussed at particular length, with participants emphasising the conflict of wanting to contribute to meaningful research but recognising that it could be triggering, and the balancing of such. Numerous studies have considered the ethics of discussing sensitive topics in the context of co-production (Amann and Sleigh 2021; Isham et al. 2019), with some arguing that the cost is too great (Oliver et al. 2019). This perspective is contested by Williams et al. (2020), who argue that shared vulnerability and uncertainty are what drives improvement, and that one of the roles of researchers within co-production is to ensure that ethical practices are implemented and adhered to in order to ensure safety. Findings from the current study echo this; whilst it is important to be aware of potential risks and be prepared to address them, researchers should not be deterred from engaging in co-production. If they are, then nothing will change.

### Limitations

Only five of the six members from the original SG were able to be recruited for this study. Thus, some elements of the co-production experience could have been missed.

All participants had previously received psychological treatment for OCD. In the wider OCD community, it is recognised that a limited proportion of individuals will receive adequate and timely support (Goodwin et al. 2002; Torres et al. 2007). This means that the findings from this paper may not be directly generalisable to the wider OCD population. However, the purpose of this paper was to evaluate the experiences of those involved in this specific attempt at co-production, so we did not seek to recruit a more broadly representative sample. Further, we intend for the study findings to aid in the development of future co-production initiatives, rather than being prescriptive.

Whilst measures were taken to help participants feel able to be honest about their experiences of co-production (e.g., data collection and analysis led by two researchers external to the SG), there is still the possibility that participants withheld some of their opinions due to the involvement of the facilitators and participation from other SG members. Future studies which endeavour to explore the experiences of co-production may want to consider focusing on a broader population, and utilising methods that offer additional anonymity (e.g., qualitative surveys).

Finally, we utilised reflexive thematic analysis on a small sample, when guidance usually recommends a minimum sample size of 6–10 participants (Braun and Clarke 2013). Interpretative Phenomenological Analysis (Smith et al. 2009) was considered as an alternative method. However, as the aim of this study was to identify and make sense of shared meanings and experiences across the participants as a group, rather than focus on individual cases (Braun and Clarke 2021b), thus, reflexive thematic analysis was deemed to be the more appropriate analytic approach. In addition, the principles of information power were applied, and it was determined that the sample size was sufficient to achieve the aim of the current study.

### Implications for research and practice

The benefits of lived experience involvement within mental health research are far reaching, providing both meaning and catharsis for those involved and vital improvements in research quality, thus translating into better treatments and outcomes for patients in the future. As expected, the extent of involvement must be weighed up against practical considerations, such as funding requirements and time restrictions. However, if resources permit, co-production represents a useful and progressive methodology.

Findings from the current study present key considerations for researchers and practitioners looking to utilise co-production. Firstly, EBLE contributors’ welfare and wellbeing are vital. EBLE who participate in co-production are placing themselves in a vulnerable position by sharing their lived experience, which may additionally require overcoming previous difficult experiences (Richmond et al. 2023). Safeguarding is therefore integral to ensuring the psychological safety of all involved, as discussions could be triggering/ difficult and distressing. It is crucial that facilitators are aware of how to implement this and are provided with adequate training to do so. Facilitators can ensure psychological safety by making themselves available during and after sessions and providing warnings for potentially triggering content. It is also important to not pressure EBLE into speaking, and that all contributions are welcomed without judgement.

Facilitators must ensure that there is a genuine sharing of power throughout (Farr 2018). This can be enabled by offering EBLE opportunities to chair meetings and set ground rules, and by communicating openly about the aims of co-production and about boundaries, including the purpose of the group. Embodiment of a positive, caring attitude also has great implications for the tone of the group, empowering EBLE to share their experiences. Where possible, taking time in advance to build relationships and trust with EBLE is beneficial for co-production.

Finally, having sessions well structured, planned, and communicated in advance was appreciated by participants in the current study, who found this highly useful. It is important for facilitators to be open to feedback and agile in incorporating such, and to be flexible around the needs and schedules of EBLE. With this, the facilitators understanding must be afforded to EBLE’s lives needing to come first. As with any group work, facilitators must work to ensure every voice is heard, as inevitably some may share more or less than others.

## Conclusion

Co-production can provide a meaningful opportunity for both researchers and individuals with lived experience to bring together their expertise for the benefit of all those involved and the wider community. Benefits can be found within the action of co-production, as well as through catharsis and the opportunity to use difficult experiences to help others. Co-production should be done with care, and with genuine consideration for power dynamics and the wellbeing of EBLE. When done effectively, co-production can be a powerful method for producing high quality and meaningful clinical research that truly serves those it is created for.

## Supplementary Information

Supplementary Material 1

## Data Availability

The datasets generated and analysed during the current study are not publicly available due preservation of confidentiality and anonymity of the participants but are available from the corresponding author on reasonable request.

## Footnotes

1 The steering group members with lived experience of OCD were consulted to determine how they wished to be collectively referred to. As a result, the term Experts By Lived Experience (EBLE) will be used.
