# Supplementary material for: “Are we genuinely going to have our voices heard?” The experience of co-producing a blended intervention to prevent relapse in obsessive-compulsive disorder: a qualitative study on the perspectives of experts by lived experience
Source: BMC Psychiatry. 2024 Dec 18;24:906. doi: 10.1186/s12888-024-06355-1 (PMC11654374; doi:10.1186/s12888-024-06355-1)
Supplement: Supplementary file 1 — Supplementary Material 1 [file 12888_2024_6355_MOESM1_ESM.docx]

**RQ: How did people with experience of OCD experience coproducing a relapse prevention intervention for OCD via a steering group?**

**Introduce self, thank participant for completing the questionnaire**

We would like to understand your experience of being a part of the steering group and the process of developing the intervention. We thought that perhaps there might be 3 different aspects that might be useful to explore:

1. The process of being involved in the **steering group** – your experience of coming along to the meetings each week? *(If further info if requested: things like your experience of the group dynamics and experience of how this was managed)*
2. The process of being involved in the method of **co-producing** an intervention
3. The process of being a part of group where you are **working with other people with personal experience of OCD**

**This is the structure the interview will take. How does that sound to you? Do you have any questions before we get started?**

**Thank you for providing your written consent for us to record this interview. The recording will be stored securely and listened back to by a member of the research team, this is to make sure the transcript is accurate for us to analyse, and that the transcript is fully anonymous. Does that sound ok?......**

**Once I have started recording, I will ask if I can please confirm your consent again for the recording**

**START RECORDING**

**Ok, so I have started recording, please can you confirm if you consent to me recording the interview today?**

To start off with I wondered if you could you tell me a bit about what originally drew you to being involved in this research? (Piqued your interest)

**Section 1 - Steering group process/ Psychological safety**

1. What was being a part of the group like for you?
2. What was your experience of sharing your views or ideas within the meeting?
3. How able did you feel to share your views, if your views were similar or different to others?
4. How did you communicate those views? (use of chat/speaking in group/email after)
5. How did you feel your views were recognised or responded to?

**Section 2 - Power/coproduction methodology:**

The method used to create the intervention was called co-production: **this is an approach in which researchers, practitioners and members of the public work together, sharing power and responsibility from the start to the end of the project, including the generation of knowledge.**

1. Can you tell me a bit about your experience of how you felt the co-production worked in this instance?
2. What are you views on the idea of co-production in terms of people with personal experience contributing to research/ developing treatments in areas they are affected by?
3. What do you think about people with personal experience **not** being involved in research or developing treatments, for the difficulties they are affected by?
4. Can you tell me a bit about how you felt power was used within the group?
5. Could you tell me a bit about your experience of being involved in this research?

**Section 3 - Experience of working with others impacted by OCD**

In co-production, we are asking steering group members to draw on their own experiences which at times may have been very difficult and or distressing, with the aim of constructively working together to develop an intervention with the potential to help others.

1. I wonder if you could tell me about how this experience was for you?
2. Perhaps, what was it like to be part of a group working alongside others affected by OCD?
3. What was it like developing an intervention with the aim of helping others affected by OCD?
4. Were there any benefits that you didn’t expect from taking part?
5. Where there any aspects that you found difficult or challenging that you hadn’t expected?
6. Did you find any of the topics discussed triggering – how did you feel that was managed?
7. Did you find anything unexpected has come out of the steering group meetings?
8. Any new techniques/therapies that you might have used to help yourself that you got from another member?
9. A new perspective to OCD?

**Future Research Recommendations:**

1. If you were aske to advise a new facilitator of a coproduction group, what would you advise them to include?
   1. What do you think makes facilitators feel approachable or unapproachable?
2. What impact, if any, do you think this research could have for the OCD community?
   1. On relapse prevention?
   2. With regard to coproduction?

**Finishing questions**

Reflecting on your own experience of being involved in coproduction research, do you think there are any potential benefits of an intervention being created through coproduction? Can you tell me about these?

Is there anything that we haven’t covered that you wished to add before we finish today?

Thank you very much for you time and input today.
